# Supplementary material for: Prediction of Prognosis, Immunotherapy and Chemotherapy with an Immune-Related Risk Score Model for Endometrial Cancer
Source: Cancers (Basel). 2023 Jul 19;15(14):3673. doi: 10.3390/cancers15143673 (PMC10377799; doi:10.3390/cancers15143673)
Supplement: Supplementary file 1 [file cancers-15-03673-s001.zip › cancers-2496400-supplementary.pdf]

Prediction of Prognosis, Immunotherapy and Chemotherapy with an Immune-related Risk Score Model in Endometrial Cancer

Wei Wei, Bo Ye, Zhenting Huang,Xiaoling Mu, Jing Qiao, Peng Zhao, Yuehang Jiang, Jingxian Wu and Xiaohui Zhan

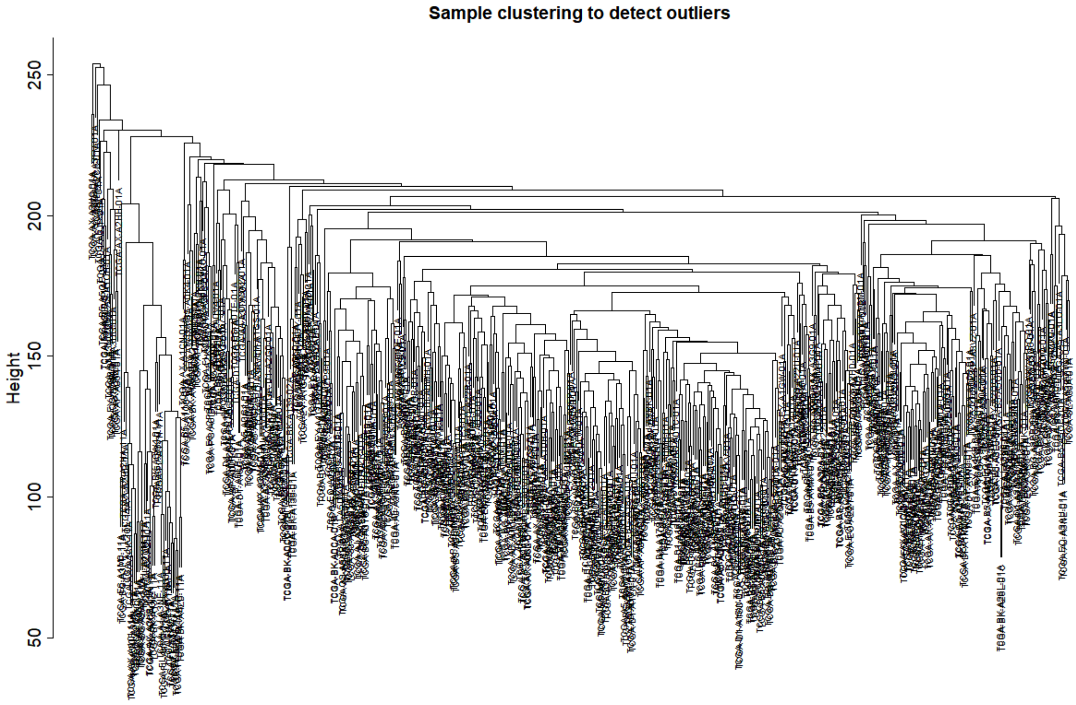

Figure S1. Sample clustering to detect outliers.

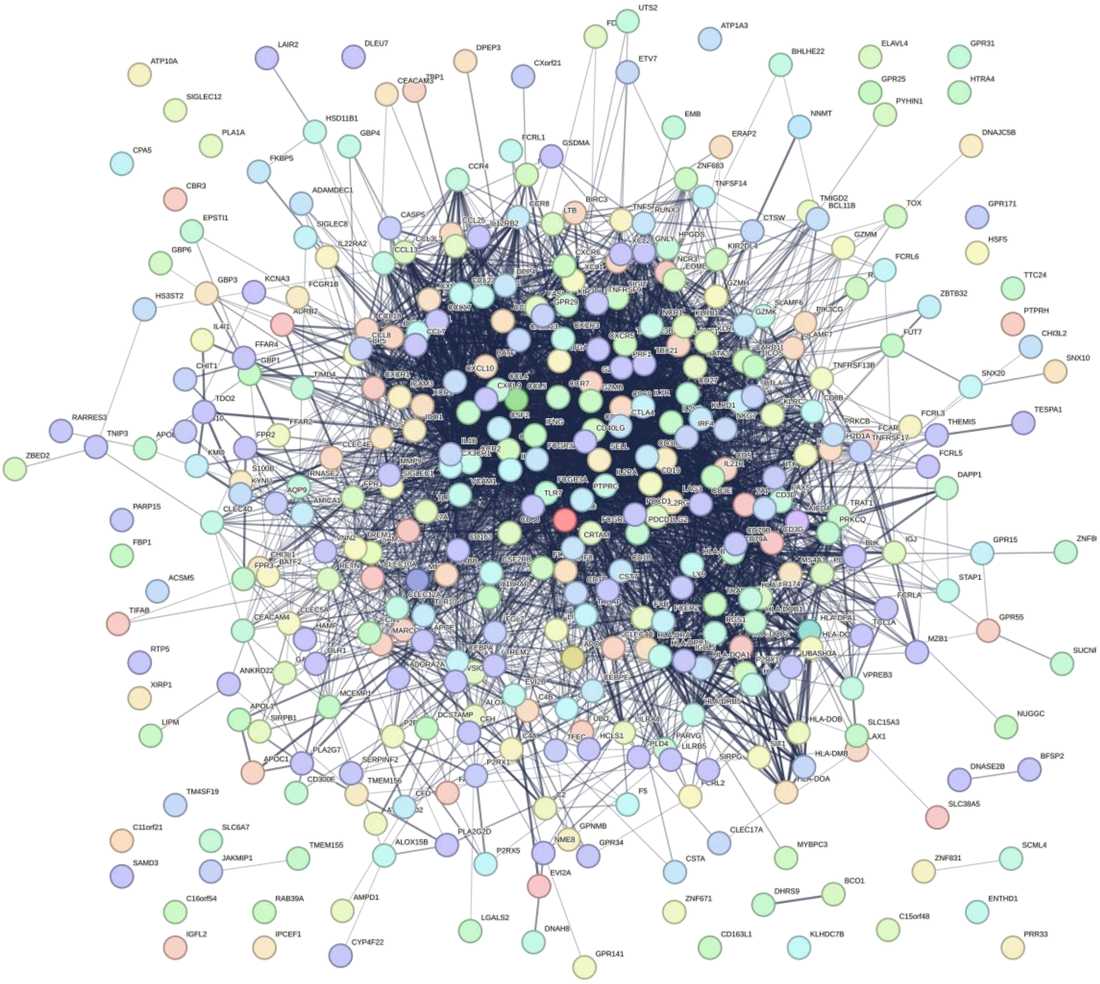

Figure S2. Protein-protein interaction (PPI) network.

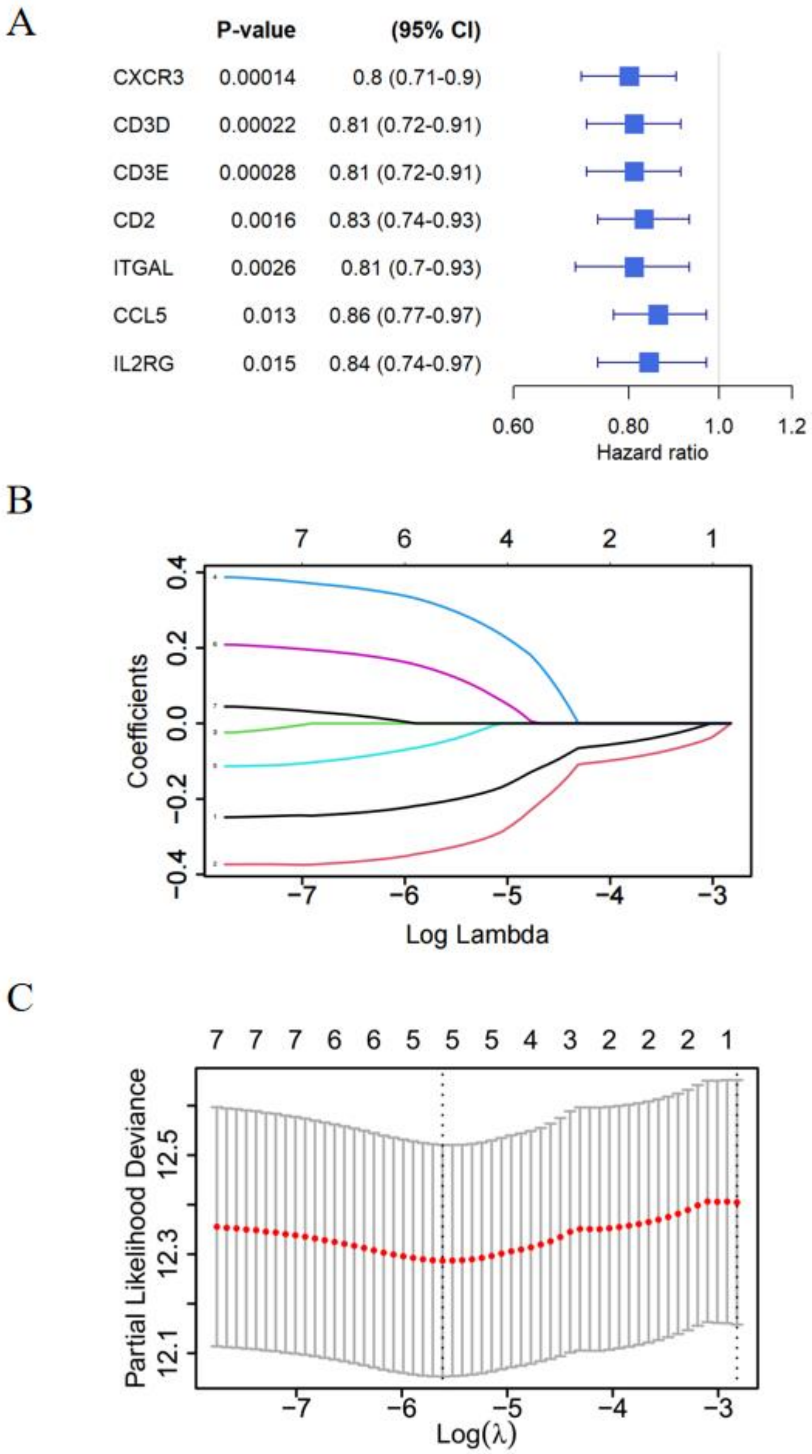

Figure S3. The construction of IRSM. (A) Univariate Cox regression analysis of immune-related key genes. (B) Lasso regression coefficient. (C) The selection of lambda for Lasso regression.
